# Prediction of Mortality and Outcome of Various Trauma Scores in Polytrauma Patients

**DOI:** 10.7759/cureus.69992

**Published:** 2024-09-23

**Authors:** Ram C Besra, Samir Toppo, Pankaj Bodra, Anmol Kujur, Marshal B Tudu, Binit Bharti, Harish Baskey, Nayan Sinha

**Affiliations:** 1 General Surgery, Rajendra Institute of Medical Sciences, Ranchi, IND; 2 Anaesthesiology, Rajendra Institute of Medical Sciences, Ranchi, IND; 3 Neurosurgery, Rajendra Institute of Medical Sciences, Ranchi, IND

**Keywords:** blunt abdominal trauma, chest trauma, injury severity score (iss), new injury severity score (niss), polytrauma, revised trauma score (rts), trauma revised injury severity score (triss)

## Abstract

Background

In developing nations, the primary cause of death is trauma, and the prevalence of trauma is increasing as more vehicles are driven. Numerous trauma scoring systems have been created in order to predict the mortality rate and patients with trauma's prognosis. The purpose of the current study was to assess the prognostic ability of various trauma scoring systems for patients' mortality and morbidity in cases involving chest and abdominal injuries, as they are common in the surgery department.

Methodology

At Ranchi, Jharkhand's Rajendra Institute of Medical Sciences, a prospective observational study was conducted from June 2021 to September 2022. All patients who met the requirements for inclusion were older than 18 and reported chest and abdominal trauma totaling 204. Before any essential therapies, primary care and resuscitation, including airway maintenance, breathing, circulation, and hemorrhage control, were established. A comprehensive clinical evaluation was done based on each patient's needs. Radiological evaluation included chest X-ray and ultrasonography (USG) for chest trauma, whereas USG (FAST) and CT scans were for abdominal trauma. Trauma scores, such as the Revised Trauma Score (RTS), the Trauma Revised Injury Severity Score (TRISS), the New Injury Severity Score (NISS), and the Injury Severity Score (ISS), were computed and examined using IBM Corp. Released 2011. IBM SPSS Statistics for Windows, Version 20.0. Armonk, NY: IBM Corp.

Results

Of the 204 patients, 14.7% were female and 85.3% were male. The age range of 21-30 years old accounted for the largest percentage of cases (28%). 50 percent of injuries were caused by automobile accidents, while 25% were the result of assaults. At 83.8% versus 16.2%, blunt injuries were substantially more common than penetrating ones. In comparison to the chest, the abdomen was more frequently involved. The study's findings demonstrated that, while every trauma scoring was statistically significant in predicting mortality, the New Injury Severity Score (NISS), as well as the Trauma Revised Injury Severity Score (TRISS), became the most effective in predicting mortality (p < 0.0001).

Conclusion

According to the results, the most precise trauma grading method for chest and abdominal injuries is the Trauma Revised Injury Severity Score (TRISS), even though all other trauma scoring systems are useful in predicting patient outcomes. Surgeons using these metrics to predict outcomes and make well-informed treatment decisions can benefit greatly.

## Introduction

The restorative perspective defines trauma as an injury to the human body, which firmly lays the foundation for it to be among the primary global causes of morbidity and mortality. Injuries of this nature can result from a myriad of factors, including road traffic accidents, falls, intentional assaults, and natural disasters [1]. Of these numerous factors, road traffic accidents are regarded as the primary global cause of injuries connected to trauma. In India, injuries are thought to be the cause of around one million fatalities annually, and approximately 20 million require hospitalization following trauma-related events [2,3]. This reiterates the need for effective trauma management strategies and accurate prognostic tools for clinical decision-making.

The developed injury scoring systems became the mainstay of injury epidemiology and are in continuous use in clinical management and research. ISS, TRISS, and updated injury severity scores are in routine use to estimate mortality risk and compare injury severity [4]. Impartial evaluation of injury is not only essential for treatment strategies but also for meaningful clinical trials. Properly operating scoring systems assist in the classification of patients according to the severity of their injuries, establish the degree of urgency necessary for their treatment, and facilitate the allocation of resources within a healthcare institution [5,6].

Without a doubt, trauma scoring frameworks have advanced over the past three decades in providing measurable and comparable standards for assessing the degree of injury [7,8]. To ascertain the extent of damage the human body has sustained, several anatomical and physiological assessment tools, including the Damage Seriousness Score, Unused Harm Seriousness Score, and the Reexamined Injury Score, have been developed [9]. These grading systems are essential for determining a chronic patient's prognosis, enhancing the triage process, and maximizing the efficiency of injury care. Therefore, anatomical ratings indicate the severity of the physical injury, while physiological scores show how the body is responding to the injury based on important changes in signs and awareness levels. In order to provide a moving-forward foundation for survival forecasts, coordinated scoring systems such as TRISS predict mortality and morbidity [10]. For example, an anatomical scoring system that assigns a score based on the extent of damage to various anatomical regions [11].

Despite being the most widely used framework globally, it has certain drawbacks, such as when patients have multiple wounds in the same body region or multiple damaged body regions [12]. By taking into account the three most serious wounds, at least in their respective areas, the NISS was deduced as a step toward the ISS in an attempt to thwart these shortages. Systolic blood pressure (SBP), respiratory rate (RR), and the Glasgow Coma Scale (GCS) are coordinated by physiological scoring frameworks like the Revised Trauma Score (RTS), which provide a trustworthy evaluation of a patient's timely physiological response to trauma [13,14]. TRISS is a useful tool for assessing included esteem within the proportion of plausible outcomes of survival to mortality of the damaged patient because it incorporates the ISS and RTS scores in calculating the possibility of surviving as the patient's age increases [15,16]. Nevertheless, despite their widespread use, the accuracy and bounds of the suitability of these scoring systems promote validation, particularly in specific patient populations, such as those with abdominal and chest trauma. The current study attempts to determine, at least in part, how trauma-scoring frameworks contribute to the prognosis of death and dismal results in patients who exhibit thoracic and abdominal injuries [17,18]. The ideal goals are to assess how well various damage score systems predict mortality and morbidity in this trauma group and to identify the limitations of various trauma scoring frameworks when surgical trauma occurs.

## Materials and methods

Study site

The ‘Rajendra Institute of Medical Sciences’ hosted the study Trauma Centre and General Surgery Division in Ranchi, a tertiary care medical facility offering multispecialty services, including treatment for committed injuries. 

Study duration

The study was conducted between June 2021 and December 2022, a period of 18 months. 

Study design

In a tertiary care institution, the current study was planned as a prospective observational study. This study's main goal was to determine which trauma grading system is most useful for predicting a patient's outcome when they have thoracic and abdominal injuries.

Sample size

There were 204 participants in this study. Based on historical statistical data regarding the number of patients in the study who were hospitalized following thoracic and abdominal injuries, the sample size was established.

Study population

Patients with thoracic and abdominal trauma only who were hospitalized in the general surgery department served as the study subjects. Patients were monitored from the time of admission till their discharge or death.

Ethical approval

The conduct of the study was authorized ethically by the Institutional Ethics Committee of the Rajendra Institute of Medical Sciences, Ranchi (memorandum no. 230, dated 19/05/2021). The Declaration of Helsinki was fully followed while conducting the study.

Inclusion criteria

The inclusion criteria include those patients who have sustained trauma to the abdomen and chest and then got admitted to the surgical ward. Participants should be eighteen years of age or older. Furthermore, the subjects must be willing to give consent in writing form to participate in the study.

Exclusion criteria

This had been excluded from the study: individuals less than 18 years old who cannot consent and show poor decision-making skills because of mental disorders and other medical conditions like cardiac diseases with anticoagulation. Head injury and pregnant patients were excluded. The researcher excluded patients with burns from the study.

Methods of measurement of outcome

Upon admission, every patient or trustworthy informant had a thorough history taken, which was followed by a thorough physical examination. Primary and secondary surveys were carried out in every case, and the clinical assessment was based on Advanced Trauma Life Support (ATLS) recommendations. Vital signs, the specifics of the injury mechanism, and the injury's location were duly documented. The ATLS recommendations served as the basis for the first attempts at resuscitation with the ABCDE component, airway maintenance, breathing and ventilation, circulation and hemorrhage control, prevention of disability from spine injury, and exposure to concealed injuries. Patients received all additional radiological examinations after hemodynamic stabilization, and they were moved to the surgical department or intensive care unit as needed. Based on the above-mentioned findings, trauma scores such as RTS, TRISS, NISS, and ISS were all computed and documented.

Patients were continuously monitored during their hospital stay, and every procedure, including the implantation of an intercostal drain and operative procedure, was meticulously documented. Other indicators of morbidity that were noted were the duration of hospital stay, the requirement for respiratory assistance, and the monitoring in the critical care unit.

Statistical analysis

IBM Corp. Released 2011. IBM SPSS Statistics for Windows, Version 20.0. Armonk, NY: IBM Corp. was used for data analysis after the data was imported into Microsoft Excel tables. Omnicalcator (Krakow, Poland) [31] was used to compute injury scores. Calculations were made using descriptive statistics, such as mean and standard deviations. An examination of correlation was carried out. To evaluate how well outcomes were predicted, ROC curves were created for every injury score. In addition to determining the accuracy of result prediction for various cut-off positions, sensitivity and specificity were also determined. In order to assess the discriminating power of each scoring system, the area under the receiver operating characteristic curve was determined: an area of 0.5 denoted chance and an area of 1.0 perfect discrimination. To calculate cut-off values that maximize sensitivity and specificity for each injury score, she supplied the Youden index. Information Analysis for each injury score system, sensitivity, specificity, and accuracy were determined for various cut-off points in order to compare their prognostic power. Without the use of any predetermined, arbitrary cut-off points, overall, ROC curves were used to compare performances. An AUROC, or area under the curve, of a given injury score indicates its dependability and discriminatory power; a higher AUROC indicates higher predictive accuracy.

## Results

Age-wise distribution of patients

Table 1 summarizes the distribution of patients by age group​. The majority of the patients, or 28.4% of the total, were between the ages of 21 and 30, with patients under 20 making up 23% of the group. These age groups may have a higher incidence of trauma because they use two-wheelers more frequently, which puts them at risk for motor vehicle crashes.

**Table 1:** Age-wise distribution of patients

Age (years)	Frequency	Percent	Valid Percent	Cumulative Percent
<20	47	23.0	23.0	23.0
21-30	58	28.4	28.4	51.5
31-40	36	17.6	17.6	69.1
41-50	35	17.2	17.2	86.3
51-60	19	9.3	9.3	95.6
>60	9	4.4	4.4	100.0
Total	204	100.0	100.0	100.0

Sex-wise distribution of patients

Table 2 indicates that, of the research population's demographic characteristics, men made up 85.3% of the cases, while women made up 14.7%. Males are more likely to be predominately involved in outdoor activities, which puts them at a larger chance of experiencing traumatic events. Although there has been an increase in trauma cases among women in industrialized nations, violence is still the leading cause of harm for women in underdeveloped nations.

**Table 2:** Patient distribution by sex

Sex	Frequency	Percent	Reliable Percentage	Total Percentage
Female	30	14.7	14.7	14.7
Male	174	85.3	85.3	100.0
Total	204	100.0	100.0	100.0

Mode of injury

As demonstrated in Table 3, road traffic accidents (RTA) were the most common way that injuries occur in the patient population, accounting for 50% of cases. At 24.5%, assaults ranked as the second most prevalent cause, followed by stab wounds (9.3%) and falls from elevation (8.3%). RTA rates are closely correlated with rising motor vehicle usage, and they have particular consequences for two-wheeler riders, who are likewise disproportionately vulnerable.

**Table 3:** Injury mode distribution

Mode of Injury	Count	Percentage	Reliable Percentage	Total Percentage
Animal Attack	7	3.4	3.4	3.4
Assault	50	24.5	24.5	27.9
Bullet Injury	9	4.4	4.4	32.4
Fall from Height	17	8.3	8.3	40.7
Road Traffic Accident (RTA)	102	50.0	50.0	90.7
Stab Injury	19	9.3	9.3	100.0
Total	204	100.0	100.0	100.0

Type of injury

As seen in Table 4, which represents 83.8% of the cases reported, blunt trauma was significantly more common than penetrating injuries. Accidents involving motor vehicles are the most common reason for blunt injuries. A violent environment was more frequently mentioned when describing penetrating injuries.

**Table 4:** Type of injury distribution

Type of Injury	Count	Percentage	Reliable Percentage	Total Percentage
Blunt	171	83.8	83.8	83.8
Penetrating	33	16.2	16.2	100.0
Total	204	100.0	100.0	100.0

Region affected

According to Table 5, the abdomen accounted for 69.6% of all cases of afflicted areas with abdominal trauma alone. Following this were trauma to the chest alone at 14.7% and trauma to the abdomen and chest combined at 15.7%. The most frequently injured body part in all blunt and penetrating injuries-especially those sustained in motor vehicle accidents-was the abdomen.

**Table 5:** Region affected distribution

Region Affected	Frequency	Percent	Valid Percent	Cumulative Percent
Abdomen	142	69.6	69.6	69.6
Abdomen + Chest	32	15.7	15.7	85.3
Chest	30	14.7	14.7	100.0
Total	204	100.0	100.0	100.0

Outcome of patients

Table 6 shows how the trauma patient outcome, 91.2% of the 204 patients in the study, were released after receiving treatment, while 8.8% of them died due to their injuries. Patients with penetrating injuries and related abdomen and chest traumas had a dismal death rate in a considerable number of instances, suggesting a somewhat unfavorable prognosis in these circumstances.

**Table 6:** Patient outcome distribution

Outcome	Frequency	Percent	Reliable Percentage	Total Percentage
Discharged	186	91.2	91.2	91.2
Death	18	8.8	8.8	100.0
Total	204	100.0	100.0	100.0

Predictive accuracy of trauma scores

Utilizing curves for the Receiver Operating Characteristic (ROC), the predictive capabilities of the Replacement Trauma Score (RTS), New Injury Severity Score (NISS), Injury Severity Score (ISS), and Trauma and Revised Trauma Score (TRISS) were assessed. The Area Under the ROC Curve (AUROC) for each of these scoring systems was ascertained as follows:

ISS: 0.8169

NISS: 0.8361

RTS: 0.7953

TRISS: 0.8521

All four trauma ratings were statistically significant in predicting death, according to the Receiver Operating Characteristic (ROC) curves; however, trauma and the Revised Trauma Score (TRISS) and New Injury Severity Score (NISS) demonstrated the highest predictive precision. 

**Figure 1:**
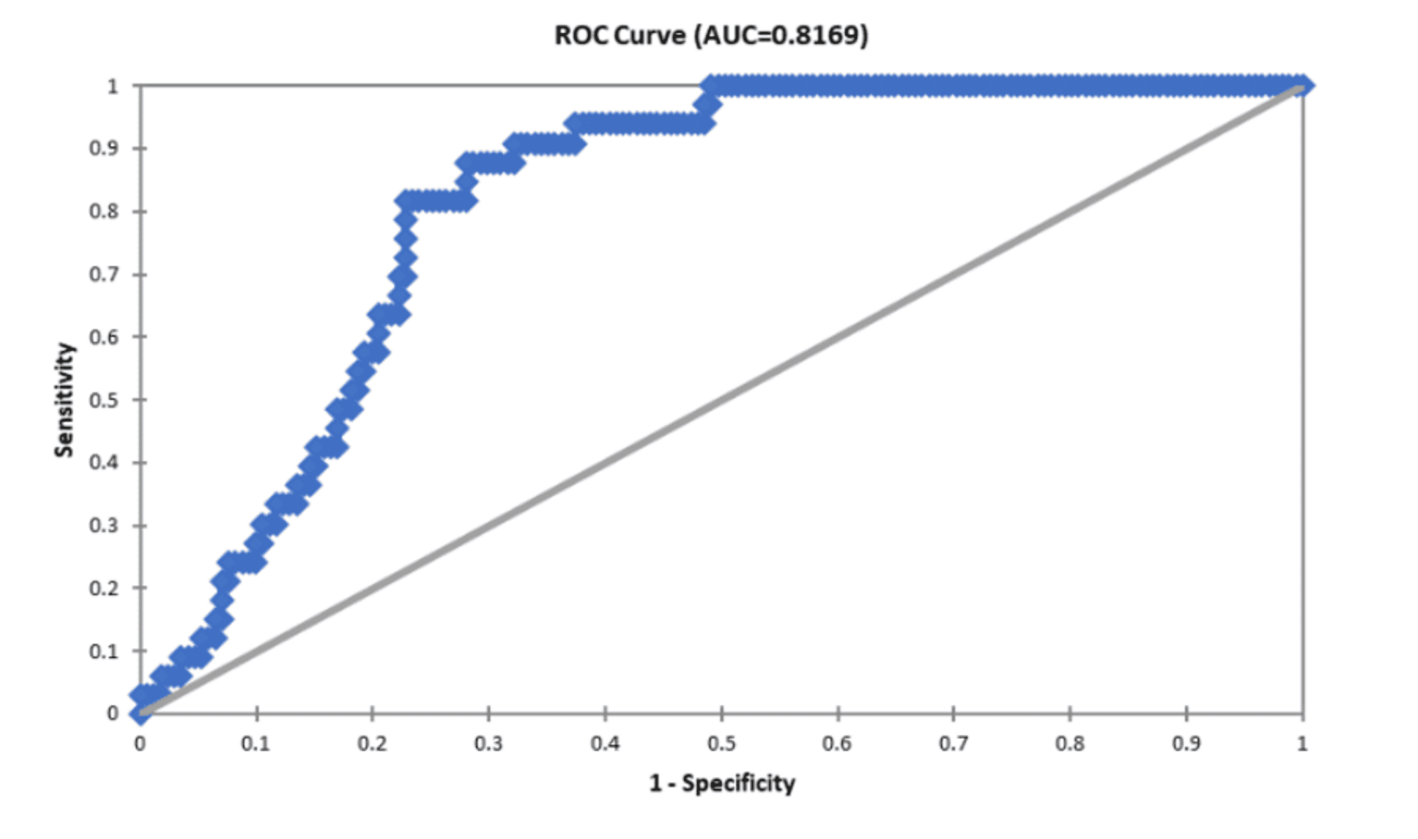
Receiver operating characteristic (ROC) curve in relation to injury severity score (ISS)

Figure 1 below represents the Receiver Operating Characteristic (ROC) curve, which represents the predictive accuracy of the Injury Severity Score (ISS) in relation to the mortality of the patients. The Area Under the Receiver Operating Characteristic curve (AUROC) for ISS is 0.8169; hence, this model has a good predictive ability to predict the outcomes of the patients based on the severity of injury. 

**Figure 2:**
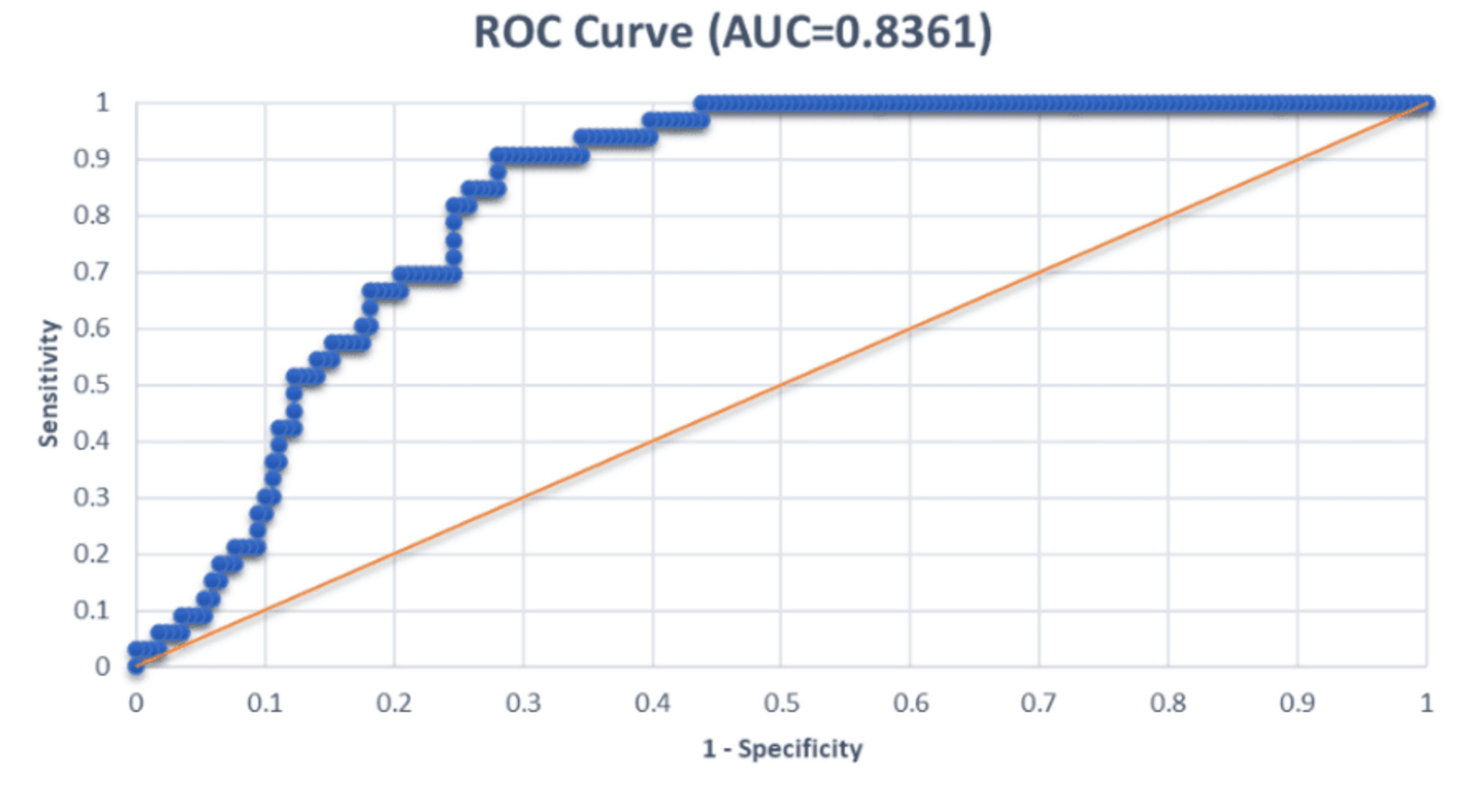
Receiver operating characteristic (ROC) curve in relation to new injury severity score (NISS)

Figure 2 depicts the receiver operating characteristic (ROC) curve for the New Injury Severity Score (NISS), representing the model performance in the prediction of mortality. The Area Under the Receiver Operating Characteristic curve (AUROC) is 0.8361 for the New Injury Severity Score (NISS), which is higher than that for the Injury Severity Score (ISS). Thus, the New Injury Severity Score (NISS) indicates a strong predictive power in whether a patient will survive or die from trauma. 

**Figure 3:**
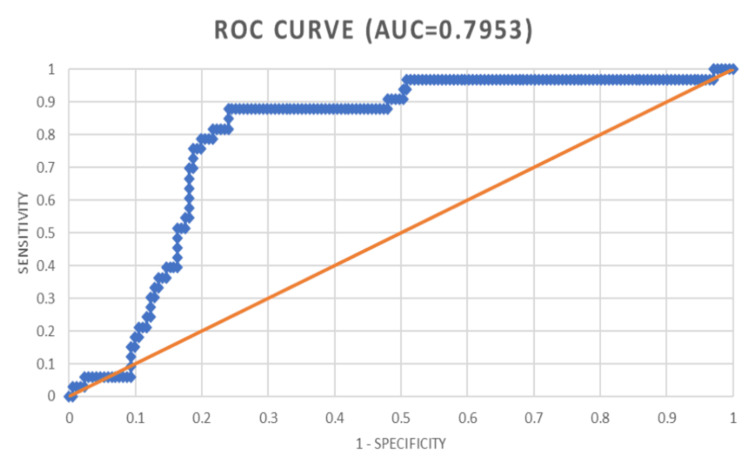
Receiver operating characteristic (ROC) curve in relation to revised trauma score (RTS)

Figure 3 presents the performance of the Revised Trauma Score (RTS) regarding mortality risk assessment on the receiver operating characteristic (ROC) curve. The area under the receiver operating characteristic curve (AUROC) of the RTS is 0.7953, demonstrating moderate predictive validity. It is effective but slightly less effective than NISS and ISS in its predictive capability. 

**Figure 4:**
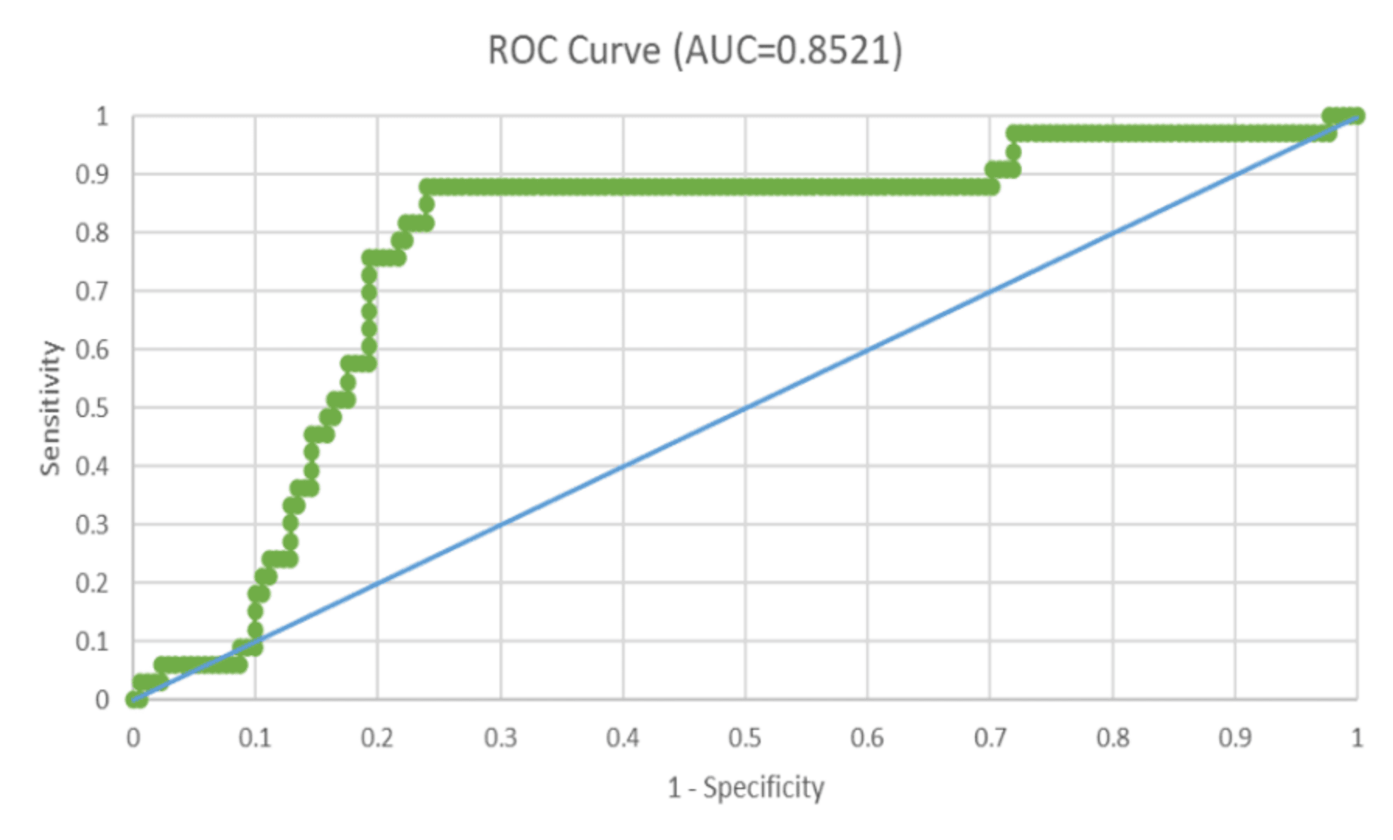
Receiver operating characteristic (ROC) curve in relation to trauma and the revised trauma score (TRISS)

The Receiver Operating Characteristic (ROC) curve of trauma and the Revised Trauma Score (TRISS) have an Area Under the Receiver Operating Characteristic curve (AUROC) of 0.8521 (Figure 4 ). TRISS, among all the trauma scores in this study, carries the highest predictive value and has excellent precision in discrimination against patient mortality after traumatic injury.

Trauma scores statistical summary

Table 7 presents a statistical summary of the trauma scores. Regarding TRISS, RTS, NISS, and ISS, the corresponding mean and standard deviation (Mean ± SD) were 15.9118 ± 19.6524, 19.6569 ± 21.7029, 7.2255 ± 1.0161, and 6.0091 ± 21.9027, respectively. The bigger mean scores for both ISS and NISS indicate that the study population's injuries were more severe.

**Table 7:** Statistical summary of trauma scores

Trauma Score	Observations	Minimum	Maximum	Mean ± SD
Injury Severity Score (ISS)	204	1.0000	75.0000	15.9118±19.6524
New Injury Severity Score (NISS)	204	1.0000	75.0000	19.6569±21.7029
Revised Trauma Score (RTS)	204	2.5418	7.8410	7.2255±1.0161
Trauma and the Revised Trauma Score (TRISS)	204	0.0030	99.6300	6.0091±21.9027

Statistical analysis of trauma scores

The statistical tests for the null hypothesis (H0: Y=0.1618) for each trauma score are shown in Table 8. The most effective indicators of mortality were NISS and TRISS, with all trauma scores demonstrating statistical significance in mortality prediction at p < 0.0001.

**Table 8:** Statistical test results for trauma scores

Trauma Score	Statistic	Degrees of Freedom (DF)	Chi-square	P-value > Chi²
Injury Severity Score (ISS)	-2 Log(Likelihood)	1	18.0135	< 0.0001
	Score	1	22.1180	< 0.0001
	Wald	1	17.7784	< 0.0001
New Injury Severity Score (NISS)	-2 Log(Likelihood)	1	28.3839	< 0.0001
	Score	1	32.7200	< 0.0001
	Wald	1	25.6407	< 0.0001
Revised Trauma Score (RTS)	-2 Log(Likelihood)	1	17.5247	< 0.0001
	Score	1	21.2540	< 0.0001
	Wald	1	16.4608	< 0.0001
Trauma and the Revised Trauma Score (TRISS)	-2 Log(Likelihood)	1	16.6565	< 0.0001
	Score	1	20.1617	< 0.0001
	Wald	1	15.8498	< 0.0001

Of the 204 patients in this study, 85.3% were male. Ages 21 to 30 made up the largest age group (28%). Automobile accidents were the cause of half of the injuries. Furthermore, 16.2% of patients had penetrating trauma, and 83.8% of patients suffered blunt trauma. The most frequently affected area was the abdomen. This study looked at the prediction accuracy among the RTS, TRISS, NISS, and ISS scores. Research has demonstrated that when it comes to predicting mortality, TRISS and NISS offer the most predictive value. All trauma ratings, but especially NISS and TRISS, were shown to be significant predictors of death by the statistical analysis results.

## Discussion

This study looked at the injury patterns and demographic traits of trauma survivors and produced data that was in line with earlier research findings. 85.3% of the study participants identified as male, indicating that the study population was predominantly male, a finding that is consistent with recent research [19].

Studies by Akköse Aydin S, Bulut M, et al. and Cevik Y, Doğan NÖ, et al. revealed that almost 65% of trauma cases were male victims [20]. It may be explained by the fact that men often have risky types of jobs or behaviors, such as driving or outdoor employment, which raise the accident rate and rate of physical injuries [21]. Meanwhile, the age group that demonstrated maximal susceptibility in the current study, namely, between 21 and 30 years, accounted for 28% of all the events. This is in line with earlier research, such as that of Baker SP and Agarwal ND, which found that individuals in their 20s, mainly young adults, had a fairly high risk of sustaining this type of injury [22].

Several factors may contribute to their susceptibility, such as more mobility, a higher propensity for risky conduct, and exposure to high-risk surroundings that enhance their chance of injury. The study also showed that assault was the second most frequent reason for injuries, following auto accidents. This aligns with the worldwide tendency that traffic, one of the main causes of injury is accidents, with research indicating that the range of injuries is 60% to 80%. Reiterating the significance of road traffic crashes in the injury ecosystem, Aydin et al. also identified other road traffic crashes as the primary source of injury [23].

The large number of traffic accidents, particularly involving minors, indicates the necessity for stronger traffic laws and road safety regulations in order to lower the frequency of these injuries [24]. 

According to the type and appearance of the injuries, abdominal injuries were the most common, and blunt injuries were far more frequent than those that penetrated. This is in line with earlier findings that blunt trauma has been reported more often, especially in auto accidents. This increasing trend of abdominal injury in this population also supports the clinical practice of careful evaluation and monitoring of patients who sustain blunt trauma because abdominal injuries can progress to critical, even fatal, processes within a very short period if unidentified and untreated [25]. The total mortality rate found in this current study was 8.8%, which is markedly lower than the 13.9% total reported in the study of Yousef Zadeh-Chabok et al. This decrease in the overall mortality rate might be due to a difference in the degree of injuries, the standard of trauma care given, and the availability of advanced medical care at different levels [26]. The trauma scores analyzed were the RTS, ISS, NISS, and TRISS. Although in this series NISS and TRISS have been found to be the most accurate trauma scores, all of them had a statistically significant effect on mortality prediction.

The means for ISS, NISS, RTS, and TRISS, along with their standard deviations in this dataset, were as follows: 15.9118 ± 19.6524, 19.6569 ± 21.7029, 7.2255 ± 1.0161, and 6.0091 ± 21.9027, respectively. The results showed that low RTS scores and high ISS and NISS values were related to high mortality rates, as in the studies by French and Yousefzadeh-Chabok et al. and Orhon et al. [27]. Sections under the operational characteristic of the receiver (ROC) curves for ISS, NISS, RTS, and TRISS were found to be 0.8169, 0.8361, 0.7953, and 0.8521, respectively. These findings are very much in accordance with the majority of "comparative research," which, in review by Aydin et al., reported that AUROCs for ISS, NISS, and TRISS were 0.907, 0.914 and 0.934, respectively, in predicting mortality.

The strong predictive value of NISS and TRISS on most patient groups, especially with thoracic and abdominal injuries, proves that these measures are useful and reliable for a clinical setting regarding the detection of the extent of damage. It has also validated the mortality and morbidity outcome predictions for patients using trauma rating systems like TRISS. In this respect, according to Guzzo et al. and Mitchell et al., the TRISS is a very sensitive and specific indicator of trauma-associated outcomes [28].

These findings are in agreement with other literature indicating that the TRISS outperforms other trauma scoring systems in major comparisons with ISS and RTS, especially when considering predictions of mortality and morbidity. This study, therefore, places great significance on the use of relevant, appropriate, accurate, and reliable trauma scoring within resource-poor countries with limited access to advanced imaging technologies. Despite occasional problems in its use, the ISS is still one of the most widely utilized scoring systems in this setting [29]. In this current study, the logistic regression analysis of the results revealed the NISS and TRISS to be major predictors of mortality, particularly in individuals with injuries to their abdomen and thoracic cavity. Investigations by N Stewart et al., who showed that TRISS was significantly predictive for mortality with an AUC value of 0.95 while ISS had 0.794 and RTS had 0.827, corroborate the findings of this research [30].

TRISS has shown a better rate of predicting morbidity and needs for ICU care. It proves an asset to planning and monitoring trauma care. The present study very clearly demonstrates, by objective evidence, the importance of trauma scoring systems in outcome prediction by patients and the criticality of appropriate focused interventions in lowering trauma over time, especially in high-risk groups. This again shows that much-improved trauma care, safer roads, and reliable scoring systems like NISS and TRISS can help in better outcomes and reduced mortality rates among trauma patients.

Limitations

Although the present study provides meaningful insight into demographic features, patterns of injury, and predictive accuracy of trauma scoring systems, several limitations must be noted. First, this represents a retrospective analysis and may therefore suffer from some selection and data collection biases. This study, however, has been single-centered; thus, generalization to other institutions or regions may not be so possible because populations and trauma care facilities differ. In addition, the study did not consider all the confounding variables including the individual's current state of health or variation in the level of pre-hospital care received.

Moreover, the absence of information about treatment protocols and the time of intervention, which is a critical factor in managing trauma-precludes a complete assessment of the efficiency of various care strategies. Finally, while the trauma scoring systems considered in this study, ISS, NISS, RTS, and TRISS, offered good predictive performance, application in resource-poor settings where advanced diagnostic tools and imagery may not always be available is sometimes difficult.

In the face of these limitations, future studies should attempt to include contributions from several centers, prospective study designs, and a wider range of variables in their models to better estimate the true contribution of trauma care and predict improved outcomes across diverse populations of injured patients.

## Conclusions

Study findings pointed out trauma scoring systems indicating patient prognosis for patients with abdominal and thoracic injuries. It is shown that all scoring systems, NISS, RTS, TRISS, and ISS proved to be statistically significant for certain death prediction elements in a 204-patient sample, but the TRISS score had the best predictive accuracy. This is a demonstration of the extent to which it provides the clinical context for the use of determining the multiple injuries and the therapeutic approach.

This study further supports the significance of these instruments in the field of trauma care by decisively demonstrating a correlation between increased mortality decreasing RTS scores and increasing ISS and NISS scores. Every system has its limitations, so even though this study showed that the TRISS was the most reliable scoring system to predict outcomes when compared to other systems that were looked at, these scores may still need to be further refined and validated in order to perform better, particularly when looking at different demographic groups. The study's findings unequivocally back up the application of TRISS as a foundational technique for treating abdominal and thoracic trauma. Additionally, they support more studies to create and enhance trauma grading instruments for broader use and improved patient outcomes.
